# Diagnosing external ventricular drain-related ventriculitis by means of local inflammatory response: soluble triggering receptor expressed on myeloid cells-1

**DOI:** 10.1186/s13054-014-0567-0

**Published:** 2014-10-20

**Authors:** Monica Gordon, Paula Ramirez, Alex Soriano, Manuel Palomo, Cristina Lopez-Ferraz, Esther Villarreal, Salome Meseguer, Maria Dolores Gomez, Carlos Folgado, Juan Bonastre

**Affiliations:** Department of Intensive Care Medicine, Hospital Universitario y Politecnico la Fe, Avda de Fernando Abril Martorell, n.106, 46026 Valencia, Spain; Centro de Investigación Biomedica En Red-Enfermedades Respiratorias (CibeRes, CB06/06/0028), Instituto de Salud Carlos III, Madrid, Spain; Infectious Medicine, Hospital Clinic, Barcelona, Spain; Department of Microbiology, Hospital Universitario y Politecnico la Fe, Valencia, Spain

## Abstract

**Introduction:**

External ventricular drainage (EVD)-related ventriculitis is one of the most severe complications associated with the use of EVDs. Establishing an early and certain diagnosis can be difficult in critically ill patients. We performed this prospective study to evaluate the usefulness of soluble triggering receptor expressed on myeloid cells-1 (sTREM-1) determination in cerebrospinal fluid (CSF) in the diagnosis of ventriculitis.

**Methods:**

A prospective observational study was conducted of 73 consecutive patients with EVD. Samples of CSF for culture, cytobiochemical analysis and sTREM-1 determination were extracted three times a week. Ventriculitis diagnosis required a combination of microbiological, cytobiochemical and clinical criteria.

**Results:**

Seventy-three consecutive patients were included. EVD-related ventriculitis was diagnosed in six patients and EVD-colonization in ten patients. Patients without clinical or microbiological findings were considered controls. The median CSF sTREM-1 was 4,320 pg/ml (interquartile range (IQR): 2,987 to 4,886) versus 266 pg/ml (118 to 689); *P* <0.001. There were no differences when comparing colonized-patients and controls. The best cut-off sTREM-1 value for the diagnosis of ventriculitis was 2,388.79 pg/ml (sensitivity 100%, specificity 98.5%, positive predictive value 85.71%, negative predictive value 100%). CSF proteins, glucose and the ratio CSF/serum glucose were also significantly different (*P* = 0.001). Serum biomarkers were not useful to diagnose EVD-related infection. These results were confirmed by a case–control study with ventriculitis patients (cases) and non-ventriculitis (control subjects) matched by age, comorbidities, severity scales and EVD duration (*P* = 0.004).

**Conclusions:**

CSF sTREM-1 was useful in the diagnosis of ventriculitis, in a similar measure to classical CSF parameters. Furthermore, CSF sTREM-1 could prove the diagnosis in uncertain cases and discriminate between EVD-colonization and infection.

## Introduction

External ventricular drains (EVD) are commonly used in critical care units for the treatment of acute obstructive hydrocephalus (subarachnoid hemorrhage, intracranial hemorrhage or brain tumors), for intracranial pressure monitoring or for the administration of intrathecal medication. Compared with other potential adverse effects, EVD-related ventriculitis is one of the most important complications associated with the use of these devices. According to the literature, the incidence rate of EVD-related ventriculitis ranges from 5% to 20% and it is mainly due to gram-positive cocci (*Staphylococcus epidermidis* or *Staphylococcus aureus*), but gram-negative bacteria such as *Pseudomonas aeruginosa* or *Acinetobacter baumannii* have also been found as causal agents. EVD-related ventriculitis has consequences not only in terms of mortality but also, more importantly, on the development of severe neurological sequelae. Therefore, an early recognition and an appropriate treatment are indispensable [1,2].

Clinical diagnosis of EVD-related ventriculitis is based on the presence of a neurological impairment, which often is not easily detectable in patients with severe brain injury. On the other hand, infectious signs such as fever or high leucocyte count could be due to other nosocomial complications [3]. Finally, the usual compartmentalization of an infection such as ventriculitis makes it unable to trigger a systemic inflammatory response expressed by serum biomarkers such as C-reactive protein or procalcitonin. Moreover, those biomarkers would be inefficient in discriminating between ventriculitis and other inflammatory situations [4,5].

Due to these factors, the study of cerebrospinal fluid (CSF) remains the key to the diagnosis of EVD-related ventriculitis. However, meningeal irritation due to the presence of blood degradation products could affect glucose, proteins and leucocyte count measurements in CSF [6,7]. The calculated cell-index in CSF could help in the diagnosis, but can fluctuate and a clear cut-off value for proven infection has not been established [2,8]. Recent studies suggest the role of different cytokines measured in CSF in the diagnosis of EVD-related ventriculitis, but this finding has not yet been validated [9]. Finally, microbiological CSF culture is nowadays the most reliable diagnostic tool, but its results can be delayed for up to 48 hours and false positive or negative results are possible.

In the last years, new molecules are being tested to help in the early diagnosis of several infections. Triggering receptor expressed on myeloid cells (TREM)-1 is one of these new biomarkers under study. TREM-1 is a transmembrane glycoprotein of the immunoglobulin superfamily, which is expressed on the surface of neutrophils, macrophages and mature monocytes when they infiltrate infected tissues. In the presence of lipopolysacharide and other microbial products, TREM-1 amplifies the synthesis of proinflammatory cytokines such as TNFα [10–12]. Its soluble form (sTREM-1) measured in plasma or other body fluids has shown good accuracy in diagnosing bacterial infections. A high serum level of sTREM-1 has been demonstrated in patients with sepsis [13] and patients with pneumonia present with high levels of sTREM-1 in bronchial lavage fluid [14,15]. Recently, Bishara [16] and Determann [17] analyzed the value of CSF sTREM-1 in patients with bacterial meningitis and proved its diagnostic usefulness and its prognostic value [16,17].

sTREM-1 has never been evaluated in central nervous system infections related to the presence of invasive devices such as EVDs. We hypothesized the usefulness of sTREM-1 determination in CSF in the diagnosis of EVD-related ventriculitis. We designed this study with the aim of evaluating the accuracy of sTREM-1 levels in CSF as a diagnostic tool for EVD-related ventriculitis.

## Methods

### Study design

This prospective observational study was conducted in the Intensive Care Unit (ICU) of Hospital Universitario y Politecnico la Fe (Valencia, Spain), a 21-bed medical ICU. We included consecutive adult patients who were admitted during a three-year period (from April 2008 to March 2011) and required the neurosurgical insertion of an EVD. Previously, informed consent was obtained from patients or from their relatives. The study was approved by the Ethical Committee for Clinical Research of the Hospital Universitario y Politecnico La Fe (Valencia, Spain).

### Data collection protocol

On admission, we recorded data related to demographics, medical history, current disease and indication for placement of EVD, type of EVD, antibiotic therapy (as surgical prophylaxis at the moment of EVD insertion or as continued treatment), the presence of other invasive devices and severity scales (Acute Physiology and Chronic Health Evaluation II (APACHE II)). Daily, we performed a clinical surveillance for the presence of any infection, which included clinical examination, analysis of temperature and hemodynamic parameters, and a blood test with leukocyte count, C-reactive protein (CRP) and procalcitonin (PCT) determination.

At admission and three times a week, we took samples of CSF for cytological and biochemical analysis, microbiological qualitative culture and sTREM-1 determination.

If any other kind of infection was suspected, samples of blood, tracheal aspirate, urine, surgical wound or other possible samples were collected for microbiological culture. The diagnosis of an infection different from EVD-related ventriculitis was made according to the Centers for Disease Control and Prevention criteria [18].

### Definitions

#### EVD-related ventriculitis and EVD colonization

According to criteria proposed by Lozier in 2002 [1], the diagnosis of EVD-related ventriculitis required (1) a positive culture of CSF, (2) a pathologic cytological and biochemical CSF analysis (leukocytosis, high levels of proteins and/or low levels of glucose) and (3) suggestive signs and symptoms, without another apparent cause (fever, neck stiffness, photophobia, altered mental status or seizures). The finding of a positive CSF culture without other clinical or cytobiochemical data of infection, and not followed by EVD-related ventriculitis development, was considered as colonization of the EVD [1].

### Study of inflammatory markers

Blood samples were centrifuged (1,500 rpm, 10 minutes) and serum was frozen at −80°C. PCT was measured by TRACE (Time-Resolved Amplified Crypate Emission) technology in a Kyptor analyzer (Brahms Diagnostica, Berlin, Germany). Measurement of CRP was performed with an immunoturbidimetric method using a commercial kit (Tina-quant CRP, Roche Diagnostics, Mannheim, Germany).

CSF samples were centrifuged (1,500 rpm, 10 minutes) to remove formed elements, and the supernatant was frozen at −80°C until required for analysis. Levels of sTREM-1 were determined by immunoassay with a combination monoclonal/polyclonal antibody of the immunoglobulin G1 (IgG1) type raised against TREM-1 (R&D Systems, Inc., Minneapolis, MN, USA).

### Statistical analysis

Patients were classified in three groups: 1) EVD-related ventriculitis; 2) EVD-colonization; and 3) controls (without microbiological findings in CSF and without clinical suspicion of EVD-related infection or colonization). Categorical variables were compared using the Chi square and the Fisher’s exact tests when appropriate. Comparison of numerical and categorical variables was performed with the Student’s t or the Mann–Whitney *U* tests when the second was dichotomic; analysis of variance (ANOVA) or the Kruskal-Wallis H test were carried out for variables with more than two categories. The statistical significance was set at 0.05 (95% confidence interval). Biomarkers were assessed (sensitivity and specificity) for their ability to diagnose EVD-related ventriculitis and EVD colonization. Data from the day coinciding with the median of EVD-days until the diagnosis of EVD-related ventriculitis were analyzed in the case of controls. Receiver operating characteristic (ROC) curves were made to determine the optimal cut-off values to diagnose EVD-related ventriculitis and to evaluate the general discriminative capacity of these indices. Results are expressed as medians with interquartile (25% to 75%) ranges (IQR) in parenthesis, unless otherwise stated.

As a result of the potential bias derived from the association between EVD-related infection risk and time of EVD duration, we also performed a case–control study with ventriculitis patients (cases) and non-ventriculitis (and also non-colonized) patients (control subjects) matched by age, comorbidities, severity scales and EVD duration to adjust ventriculitis and non-ventriculitis patients for these parameters.

All recorded data were analyzed using the statistical program SPSS 17.0.

## Results

### Description of the population

Seventy-three patients who required the insertion of an EVD were admitted to our ICU and all of them were included in the study. The main reasons for ICU admission were subarachnoid hemorrhage (42.5%) or intracranial hemorrhage (46.6%). A secondary intraventricular hemorrhage was present in 92% of cases. The mean score on the APACHE II scale on the day of admission to the ICU was 20.3 ± 6.8. EVD-related ventriculitis was diagnosed in six patients (8.2%) and EVD-colonization was detected in ten patients (13.7%). The median of EVD-days until infection or colonization was 11 days (IQR: 8 to 15.5) and 9 days (IQR: 6.75 to 9.25), respectively. Basal characteristics of the study population are described in Table 1.

**Table 1:** **Patient’s characteristics at Intensive Care Unit admission**

**Variable**	**All cases**	**Ventriculitis**	**Colonization only**	**Controls**	***P***
	**(number = 73)**	**(number = 6)**	**(number = 10)**	**(number = 57)**	
Age	61 ± 12.2	59.3 ± 14.4	65 ± 11.5	60.46 ± 12.15	0.873
Male sex	37 (50.7)	2 (33.3)	5 (50)	30 (52.6)	0.667
Comorbidities					
Hypertension	42 (57.5)	4 (66.7)	6 (60)	32 (56.1)	0.872
Diabetes mellitus	6 (8.2)	0 (0)	1 (10)	5 (8.8)	0.740
Dyslipidemia	25 (34.2)	3 (50)	3 (30)	19 (33.3)	0.683
Smoking	15 (20.5)	2 (33.3)	0 (0)	13 (22.8)	0.186
CIC	9 (12.3)	0 (0)	0 (0)	9 (15.8)	0.237
Previous CVA	13 (17.8)	2 (33.3)	2 (20)	9 (15.8)	0.554
CRI	2 (2.7)	0 (0)	0 (0)	2 (3.5)	0.749
Hepatopathy	2 (2.7)	0 (0)	0 (0)	2 (3.5)	0.749
Neoplasm	3 (4.1)	0 (0)	0 (0)	3 (5.3)	0.645
Immunodeficiency	2 (2.7)	1 (16.7)	0 (0)	1 (1.8)	0.088
APACHE II	20.3 ± 6.8	20.5 ± 6.7	19.3 ± 8’6	20.48 ± 6.58	0.675
Neurological pathology					0.051
SAH	31 (42.5)	3 (50)	5 (50)	23 (40.4)	
ICH	34 (46.6)	3 (50)	2 (20)	29 (50.9)	
Primary IVH	5 (6.8)	0 (0)	1 (10)	4 (7)	
Acute ischemic stroke	2 (2.7)	0 (0)	2 (20)	0 (0)	
Neoplasm	1 (1.4)	0 (0)	0 (0)	1 (1.8)	
Secondary IVH	67 (92)	6 (100)	7 (70)	54 (94.7)	0.024
Type of EVD					0.022
Standard (PVC)	37 (50.7)	3 (50)	6 (60)	28 (49.1)	
Antimicrobial impregnated	31 (42.5)	2 (33.3)	3 (30)	26 (45.6)	
Silver-impregnated	1 (1.4)	1 (16.7)	0 (0)	0 (0)	
Unknown	4 (5.5)	0 (0)	1 (10)	3 (5.3)	
Prophylactic antibiotic therapy	48 (66)	5 (83)	9 (90)	34 (60)	0.112
EVD duration (days)	9.6 ± 5.3	14.8 ± 3.8	11.3 ± 4.9	8.8 ± 5.12	0.019
Other nosocomial infections					0.193
Tracheitis	8 (11)	1 (16.7)	1 (10)	6 (10.5)	
VAP	16 (22)	3 (50)	5 (50)	8 (14)	
CRB	2 (2.7)	0 (0)	0 (0)	2 (3.5)	
UTI	2 (2.7)	0 (0)	1 (10)	1 (1.8)	
ICU mortality	27 (37)	4 (66.7)	1 (10)	22 (38.6)	0.065
30-day mortality	32 (43.8)	4 (66.7)	5 (50)	23 (38.6)	0.426

Data expressed as mean ± standard deviation or number (%), unless otherwise specified. APACHE II, Acute Physiology and Chronic Health Evaluation II: *CIC,* chronic ischemic cardiomyopathy; *CRB*, catheter related bacteremia; *CRI*, chronic renal insufficiency; *CVA*, cerebrovascular accident; *EVD*, external ventricular drainage; *ICH*, intracerebral hemorrhage; *IVH*, intraventricular hemorrhage; *ICU*, intensive care unit; *PVC*, polyvinyl chloride; *SAH*, subarachnoid hemorrhage; *UTI,* urinary tract infection; *VAP*, ventilator-associated pneumonia.

Median CSF sTREM-1 initial value was 279.3 pg/ml (IQR: 143.6 to 843.8), without statistically significant differences among patients who finally developed EVD-ventriculitis, colonization or controls: 284 pg/ml (IQR: 165 to 930), 194.4 pg/ml (159.8 to 322.4) and 288.6 (IQR: 127.9 to 907.4), respectively; *P* = 0.554.

The main characteristics of EVD-related ventriculitis cases are depicted in Table 2. The EVD was replaced in all cases. All patients received parenteral antibiotic therapy and four of them also received intrathecal treatment with amikacin or colistin as adjuvant therapy.

**Table 2:** **External ventricular drainage-related ventriculitis cases**

**Case**	**Etiology**	**Preceded by colonization**	**GCS**	**Serum leucocytes (mm** ^**3**^ **)**	**Serum PCT (ng/ml)**	**CSF neutrophils (mm** ^**3**^ **)**	**CSF glucose (mg/dl)**	**CSF proteins (mg/dl)**	**CSF sTREM-1 (pg/ml)**	**ICU-mortality**
1	*A. Baumannii*	No	4	20,700	0.2	1,200	8	500	5,532	No
2	*K. pneumoniae*	No	3	17,800	0.24	*	*	*	4,670	Yes
3	*S. epidermidis*	No	7	8,100	0.1	6,500	11	552	2,513	Yes
4	*S. marcenses*	Yes	10	7,600	0.41	1,200	0	765	4,584	Yes
5	*P. aeruginosa*	No	6	15,200	0.16	215	2	277	3,146	Yes
6	*E. cloacae*	Yes	7	15,100	0.72	4,076	28	405	4,056	No

*Data of CSF biochemistry corresponding to patient 2 could not be obtained because of low values of intracranial pressure. *CSF*, cerebrospinal fluid; *GCS*, Glasgow Coma Scale; *ICU*, intensive care unit; *PCT*, procalcitonin; *sTREM-1*, soluble triggering receptor expressed on myeloid cells-1.

### Microbiological findings

Most cases of EVD-related ventriculitis were due to Gram-negative bacteria (*A. baumannii, Klebsiella pneumoniae, Serratia marcenses, P. eruginosa* or *Enterobacter cloacae*) and only one case due to *S. epidermidis*. Two of these cases were preceded by colonization of the EVD by the same microorganism (Table 2).

EVD-colonization not followed by EVD-ventriculitis was diagnosed in 10 patients, mainly due to coagulase-negative *Staphylococci* (60%) and the rest of the cases due to *Corynebacterium jeikeium, A. baumannii*, *Escherichia coli* and *Alternaria alternata*.

### EVD-related ventriculitis diagnosis

Values of the different biomarkers used in the diagnosis of EVD-related ventriculitis are shown in Tables 2 and 3.

**Table 3:** **Comparison between different markers in the diagnosis of external ventricular drainage-related ventriculitis**

**Variable**	**Ventriculitis**	**Colonization only**	**Controls**	***P***
	**(number = 6)**	**(number = 10)**	**(number = 57)**	
Temperature (°C)	38 (37.35 to 39)	37.8 (37.2 to 38)	37 (36.8 to 38)	0.053
Serum leucocytes (mm^3^)	15,150 (7,975 to 18,525)	13,900 (10,350 to 14,600)	11,500 (10,000 to 15,650)	0.741
Serum PCT (ng/ml)	0.32 (0.18 to 0.64)	0.17 (0.1 to 0.34)	0.16 (0.10 to 0.37)	0.407
Serum CRP (mg/dl)	122.5 (54.7 to 227)	258 (97 to 294)	52 (22 to 102)	0.018
CSF leucocytes (mm^3^)	4,480 (2,988 to 4,886)	13.5 (3 to 643)	89 (8.5 to 629.5)	0.007
CSF neutrophils (mm^3^)	1,200 (707 to 5,288)	16.5 (0 to 993)	102.6 (12 to 610)	0.023
CSF proteins (mg/dl)	500 (341 to 658.5)	23 (19 to 88)	71 (39 to 154)	0.004
CSF glucose (mg/dl)	8 (1 to19.5)	72.5 (59 to 107.5)	76 (61 to 88)	0.001
Ratio CSF/serum glucose	0.083 (0.008 to 0.11)	0.62 (0.57 to 0.67)	0.63 (0.47 to 0.75)	0.001
CSF sTREM-1 (pg/ml)	4,320 (2,987 to 4,886)	162.37 (105 to 406.8)	268 (117.7 to 724)	<0.001

Data expressed as median (interquartile range). *CRP*, C-reactive protein; *CSF*, cerebrospinal fluid; *PCT*, procalcitonin; *sTREM-1*, soluble triggering receptor expressed on myeloid cells-1.

In patients with ventriculitis, median serum PCT at diagnosis was 0.32 ng/ml (IQR: 0.18 to 0.64) versus 0.16 ng/ml (IQR: 0.1 to 0.36) for the rest of the patients; *P* = 0.195 and the median serum CRP was 122.5 mg/dl (IQR: 54.7 to 227) versus 65 mg/dl (IQR: 23 to 150.2); *P* = 0.344.

In infected patients, the median of glucose in CSF was 8 mg/dl (IQR: 1 to 19.5) versus 76 mg/dl (IQR: 61 to 88) in non-infected patients; *P* <0.001. The median ratio CSF/serum glucose was 0.083 (0.008 to 0.11) versus 0.627 (0.466 to 0.708); *P* <0.001. There was no difference in the classical cut-off point established for the diagnosis of bacterial meningitis (ratio CSF/serum glucose <0.6) (P = 0.057) and the best cut-off point in our sample was 0.21 (sensitivity and specificity 100%). When measured three days before the diagnosis, the ratio CSF/serum glucose did not show differences (median ratio 0.549 (IQR: 0.41 to 0.723) versus 0.59 (IQR: 0.466 to 0.708); *P* = 0.533). The median of proteins in CSF was 500 mg/dl in patients with EVD-related ventriculitis (IQR: 341 to 658.5) versus 70.5 in the rest of the patients (IQR: 25.5 to 145.25); *P* = 0.001. The classical cut-off point for bacterial meningitis (CSF proteins >50 mg/dl) did not show differences (*P* = 0.074) between the groups and the best cut-off point in our sample was 267.6 mg/dl (100% sensitivity and 99.87% specificity). There were no differences three days before the diagnosis (median 62 mg/dl (IQR: 43.75 to 104.25) versus 68 mg/dl (IQR: 44 to 128.9); *P* = 0.688). In most cases, CSF was intensely bloody and a cell-index could not be calculated.

**Figure 1:**
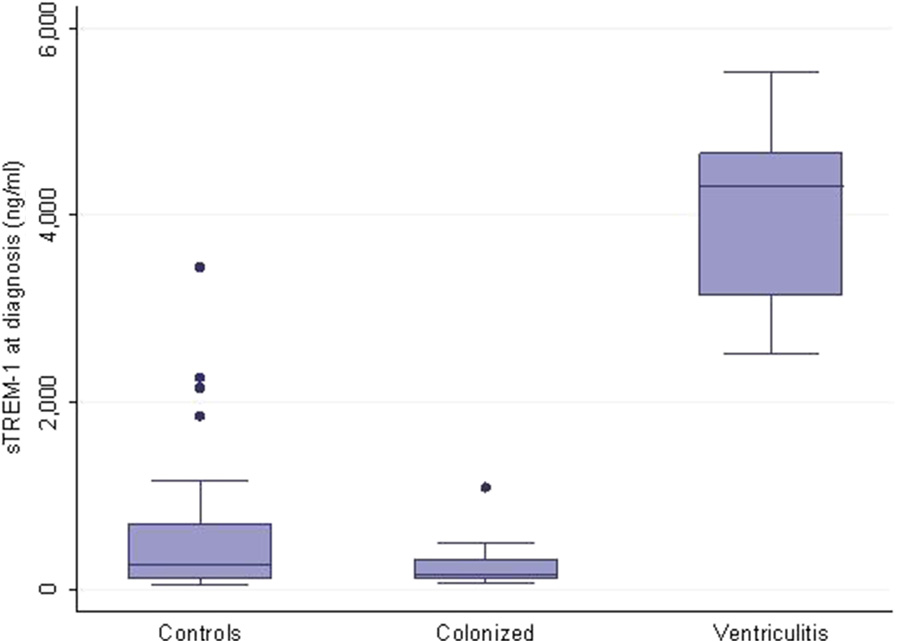
**Value of sTREM-1 in the diagnosis of external ventricular drainage-related ventriculitis.**

**Figure 2:**
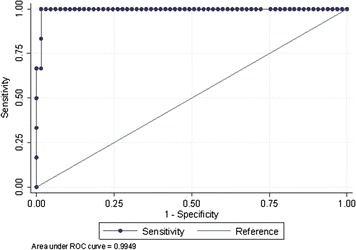
**Receiver-operating characteristic curve for cerebrospinal fluid levels of sTREM-1 in the diagnosis of external ventricular drainage-related ventriculitis.**

The median CSF sTREM-1 at the moment of ventriculitis diagnosis was 4,320 pg/ml (IQR: 2,987 to 4,886) versus 266 pg/ml (IQR: 118 to 689) in the rest of the cases; *P* <0.001. Patients with intraventricular hemorrhage were analyzed separately and the results were similar: median CSF sTREM-1 4,320 pg/ml (IQR: 2,987 to 4,886) in patients with EVD-related ventriculitis and intraventricular hemorrhage (100%), 307.58 pg/ml (IQR: 144.7 to 644.4) in patients with EV-colonization and intraventricular hemorrhage (70%) and 277.19 pg/ml (IQR: 118 to 720.5) in controls with intraventricular hemorrhage (94.7%); *P* <0.001. There were no differences between cases due to gram-positive versus gram-negative bacteria (2,513.8 pg/ml versus 4,584.4 pg/ml (IQR: 3,601 to 5,101.4); *P* = 0.143). Figure 1 shows the results for the three different groups (EVD-related ventriculitis, EVD-colonization and controls). The best cut-off point of sTREM-1 in CSF for the diagnosis of EVD-related infection was 2,388.79 pg/mL (sensitivity 100%, specificity 98.5%, positive predictive value (PPV) 85.71%, negative predictive value (NPV) 100% and area under the curve (AUC) 0.995) (Table 4 and Figure 2).

**Table 4:** **Sensitivity, specificity, positive predictive value, and negative predictive value of diagnostic tests in the diagnosis of external ventricular drainage-related ventriculitis**

**Variable**	**AUC**	**Optimum cut-off point**	**Sensitivity (%)**	**Specificity (%)**	**PPV (%)**	**NPV (%)**	**LR +**	**LR-**
Temperature (°C)	0.699	37.7	83.3	65.7	17.86	97.78	2.43	0.25
Serum PCT (ng/ml)	0.7	0.165	75	52	10.71	96.43	1.56	0.48
Serum CRP (mg/dl)	0.674	59	66.7	44.4	7.41	95.24	1.20	0.75
Serum leucocytes (mm^3^)	0.580	15,000	66.7	74.2	19.05	96.08	2.49	0.45
CSF leucocytes (mm^3^)	0.904	1,320	80	83.9	30.77	97.72	4.98	0.24
CSF neutrophils (mm^3^)	0.864	1,041	80	83	33.33	97.50	4.70	0.24
CSF glucose (mg/dl)	0.996	28.5	100	98.2	83.33	100	55	-
CSF proteins (mg/dl)	0.918	267.6	100	87.5	41.67	100	8	-
CSF sTREM-1 (pg/ml)	0.995	2,388.79	100	98.5	85.71	100	66	-

*AUC*, area under curve; *CRP*, C-reactive protein; *CSF*, cerebrospinal fluid; *LR +*, positive likelihood ratio; *LR-*, negative likelihood ratio; *NPV*, negative predictive value; *PCT*, procalcitonin; *PPV*, positive predictive value; *sTREM-1*, soluble triggering receptor expressed on myeloid cells-1.

Three days before the diagnosis, the median of sTREM-1 in CSF was 486.2 pg/ml (IQR: 202 to 532.6) in infected patients versus 292.9 pg/ml (IQR: 126 to 603) in the rest of the cases (*P* = 0.423). Cases of EVD-related ventriculitis preceded by colonization did not show a trend in the value of sTREM-1 in CSF (167 pg/ml (IQR: 136 to 197) versus 486 pg/ml (IQR: 339 to 518) in ventriculitis not preceded by colonization; *P* = 0.064).

Values of CSF sTREM-1 and other biomarkers were similar between patients with EVD-colonization and controls (Table 3). Data related to serum PCT and leukocytes or CSF sTREM-1, leukocytes, glucose and proteins were analyzed by means of the Mann Whitney *U* test and no differences were found (*P* = 0.932, *P* = 0.550, *P* = 0.459, *P* = 0.317, *P* = 0.747 and *P* = 0.176, respectively).

### Case–control study

The case–control study confirmed the results. The six cases of EVD-related ventriculitis and the ten cases of EVD-colonization were successfully matched by clinical data, severity scales and time of EVD duration with six and ten controls, respectively. The median value of sTREM-1 in CSF was significantly higher in EVD-related ventriculitis cases (4,320.2 pg/ml (IQR: 2,987.9 to 4,886) versus 745.75 pg/ml (IQR: 261.25 to 997.9); *P* = 0.004). There were no differences in the CSF sTREM-1 value when comparing EVD-colonization and controls (231.24 pg/ml (IQR: 98.4 to 452.7) versus 372.67 pg/ml (IQR: 165.83 to 752.7); *P* = 0.336).

### Prognostic value of sTREM-1

The global mortality rate due to the neurological pathology was 37% and increased to 43.8% 30 days after ICU discharge. Initial values of sTREM-1 in CSF were higher in patients who later died (670 pg/ml (IQR: 242.7 to 1229.5) versus 202 pg/ml (IQR: 113 to 562.6); *P* = 0.001).

Four of the six EVD-related ventriculitis patients eventually died; there were no statistically significant differences in the CSF sTREM-1 value (median of sTREM-1 at the moment of diagnosis in non-survivors versus survivors 3,728 pg/ml versus 4,794 pg/ml; *P* = 0.355).

## Discussion

Our findings show the usefulness of CSF sTREM-1 in the diagnosis of EVD-related ventriculitis and its capacity to discriminate between EVD-colonization and infection, in a similar measure to the classical CSF parameters. Surveillance CSF cultures were useful to detect bacterial colonization preceding the infection in one third of the cases, but sTREM-1 levels did not predict the development of EVD-related ventriculitis in any patient. In addition, higher values of sTREM-1 were related to the overall survival rate but did not show efficacy as a marker of the infection, probably because of the sample size.

There is scarce information about sTREM-1 behavior in CSF. sTREM-1 values in our series were much higher than those detected in previous studies. Determann *et al*. [16] found a median of sTREM-1 in CSF of 82 pg/ml (range: 0 to 988) in patients with bacterial meningitis versus 0 pg/ml (range: 0 to 36) in controls. Bishara *et al*. [17] obtained higher values (median 128 pg/ml, range: 0 to 484 pg/ml) in patients with bacterial meningitis. As proposed by Schade *et al*. [6], our higher values of sTREM-1 could be the result of the inflammatory reaction secondary to the presence of an intraventricular hemorrhage and the consequent blood degradation process, which was named ‘aseptic meningitis’. In fact, 92% of our patients presented with an intraventricular hemorrhage at the moment of ICU admission.

According to previous studies, CSF pleocytosis and other commonly analyzed CSF parameters, such as glucose or proteins, were not reliable as markers of infection [6,7]. However, in our study the determination of proteins and glucose in CSF and the ratio CSF/serum glucose showed statistically significant differences among those patients with EVD-related infection. Interestingly, the diagnostic cut-off points for CSF parameters were clearly different from those classically established for bacterial meningitis.

Most authors define EVD-related ventriculitis based only on the presence of a positive bacterial CSF culture (4, 5, 7). If patients in our series had been classified according to this criteria, CSF sTREM-1 values would have failed to distinguish between patients with or without positive CSF cultures: 498.6 pg/ml (IQR: 118.4 to 4,056) versus 266 pg/ml (IQR: 117 to 707), respectively; *P* = 0.074. These findings suggest a different inflammatory pattern in EVD-related ventriculitis and EVD-colonization and reinforce the need to use a combination of criteria to improve the diagnosis of infection and reduce the number of false positives.

Previous studies have shown conflicting results on the ability of serum biomarkers to identify EVD-related ventriculitis. Berger *et al*. [4] found significantly higher values of serum procalcitonin in patients with ventriculitis (4.7 versus 0.2 ng/mL). On the other hand, Martinez *et al*. [5] obtained poor results (77% specificity and 68% sensitivity) for a cut-off value of ≥1 ng/ml in patients with ventriculitis. Like these authors, in our series systemic inflammatory biomarkers were unable to diagnose the infection. The lack of accuracy of serum biomarkers in the diagnosis of localized infections has been shown [19]. Luzzani *et al*. studied 800 critically ill patients and showed a clear contrast in serum procalcitonin between localized infections (1.3 μg/L (0.6 to 2)) and those infections with systemic involvement (3.1 μg/L (1.4 to 5.2)) (sepsis, severe sepsis or septic shock).

The significance of sTREM-1 in CSF as a prognostic marker for central nervous system infection is under discussion. Determann *et al*. [16] found higher values in non-survivors (151 versus 73 pg/ml, *P* = 0.02) while Bishara *et al*. [17] found higher values among patients who survived (129 versus 35 pg/ml, *P* = 0.219). In our sample, we found an association between higher initial values of sTREM-1 and the overall survival rate; but not in the subgroup of patients with EVD-related ventriculitis.

Our study has several limitations. First, the low incidence of EVD-related ventriculitis in our study precludes drawing outright conclusions. Nevertheless, this low incidence is related to the strict EVD-associated ventriculitis diagnostic criteria that we chose. According to the criteria of the Centers for Disease Control and Prevention (18), the diagnosis of ventriculitis can be based only on a positive CSF culture. However, we have used the criteria proposed in 2002 by Lozier *et al*. [1] (including clinical and biochemical CSF data) to reduce the possibility of false positives and improve the accuracy of the diagnosis of ventriculitis. The small sample size could also affect the validity of our case–control study, but results are consistent with the rest of the statistical analysis. In contrast with previous series, we found a predominance of EVD-related ventriculitis due to gram-negative microorganisms. Our data suggest that results could be extrapolated to gram-positive cocci EVD-related ventriculitis, but further studies are needed. Serum sTREM-1 levels were not measured; however, most studies have failed to demonstrate a systemic inflammatory response to EVD-related ventriculitis [2,4].

## Conclusions

In conclusion, CSF sTREM-1 measurement has shown excellent results as a diagnostic tool for EVD-related ventriculitis but in a similar measure to the classical CSF parameters. However, CSF sTREM-1 could reinforce or even prove the diagnosis in uncertain cases. Furthermore, CSF sTREM-1 could be of great interest in validating the diagnosis of colonization versus infection. Finally, CSF sTREM-1 seems to identify those patients with severe brain injury with a worst prognosis.

### Key messages

- The best way to make an appropriate diagnosis of ventriculitis is uncertain.
- We used a combination of microbiological, cytobiochemical and clinical criteria.
- Classical CSF parameters were useful to guide the diagnosis of ventriculitis.
- sTREM-1 has an excellent diagnostic accuracy in the detection of ventriculitis.
- Ventriculitis and colonization showed differences in CSF inflammatory patterns.

## Abbreviations

APACHE II: Acute Physiology and Chronic Health Evaluation II
AUC: area under the curve
CIC: chronic ischemic cardiomyopathy
CRB: catheter related bacteremia
CRI: chronic renal insufficiency
CRP: C-reactive protein
CSF: cerebrospinal fluid
CVA: cerebrovascular accident
EVD: external ventricular drainage
GCS: Glasgow Coma Scale
ICH: intracerebral hemorrhage
ICU: intensive care unit
IgG1: immunoglobulin G 1
IQR: interquartile range
IVH: intraventricular hemorrhage
LR+: positive likelihood ratio
LR-: negative likelihood ratio
NPV: negative predictive value
PCT: procalcitonin
PPV: positive predictive value
PVC: polyvinyl chloride
ROC: receiver operating characteristic
SAH: subarachnoid hemorrhage
sTREM-1: soluble triggering receptor expressed on myeloid cells-1
TNFα: tumor necrosis factor
TRACE: Time-Resolved Amplified Crypate Emission
UTI: urinary tract infection
VAP: ventilator-associated pneumonia
